# Supplementary figures and images for: Cardiac electrical abnormalities in a mouse model of left ventricular non-compaction cardiomyopathy
Source: PLoS One. 2025 May 7;20(5):e0314840. doi: 10.1371/journal.pone.0314840 (PMC12058163; doi:10.1371/journal.pone.0314840)

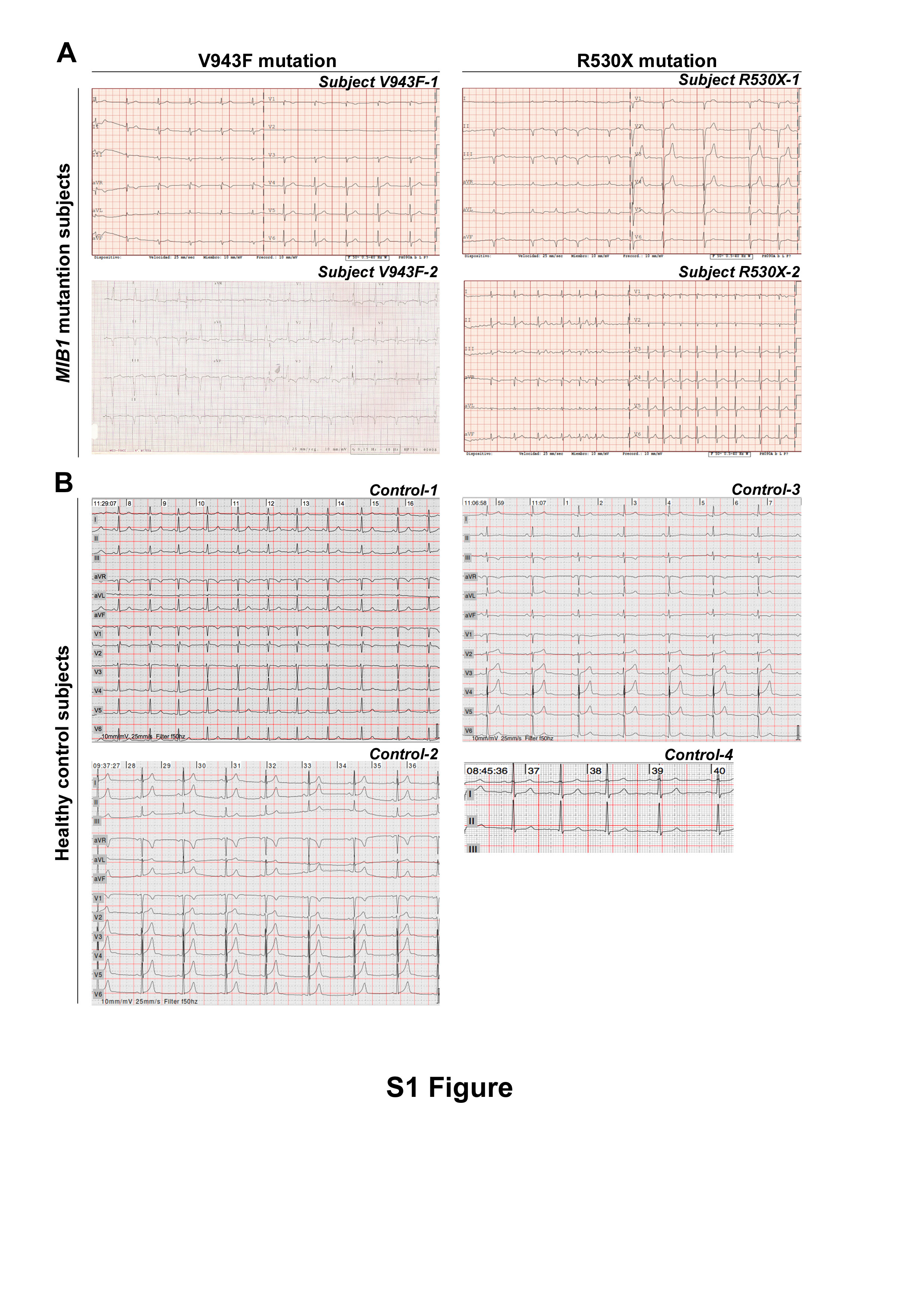

Supplement: S1 Fig — (A) Original ECG recordings from 4 individuals presenting MIB1VF943F (2 individuals) and MIB1R530X mutations (2 individuals). (B) Original ECG recordings from 4 healthy relatives. (TIF) [file pone.0314840.s001.tif]

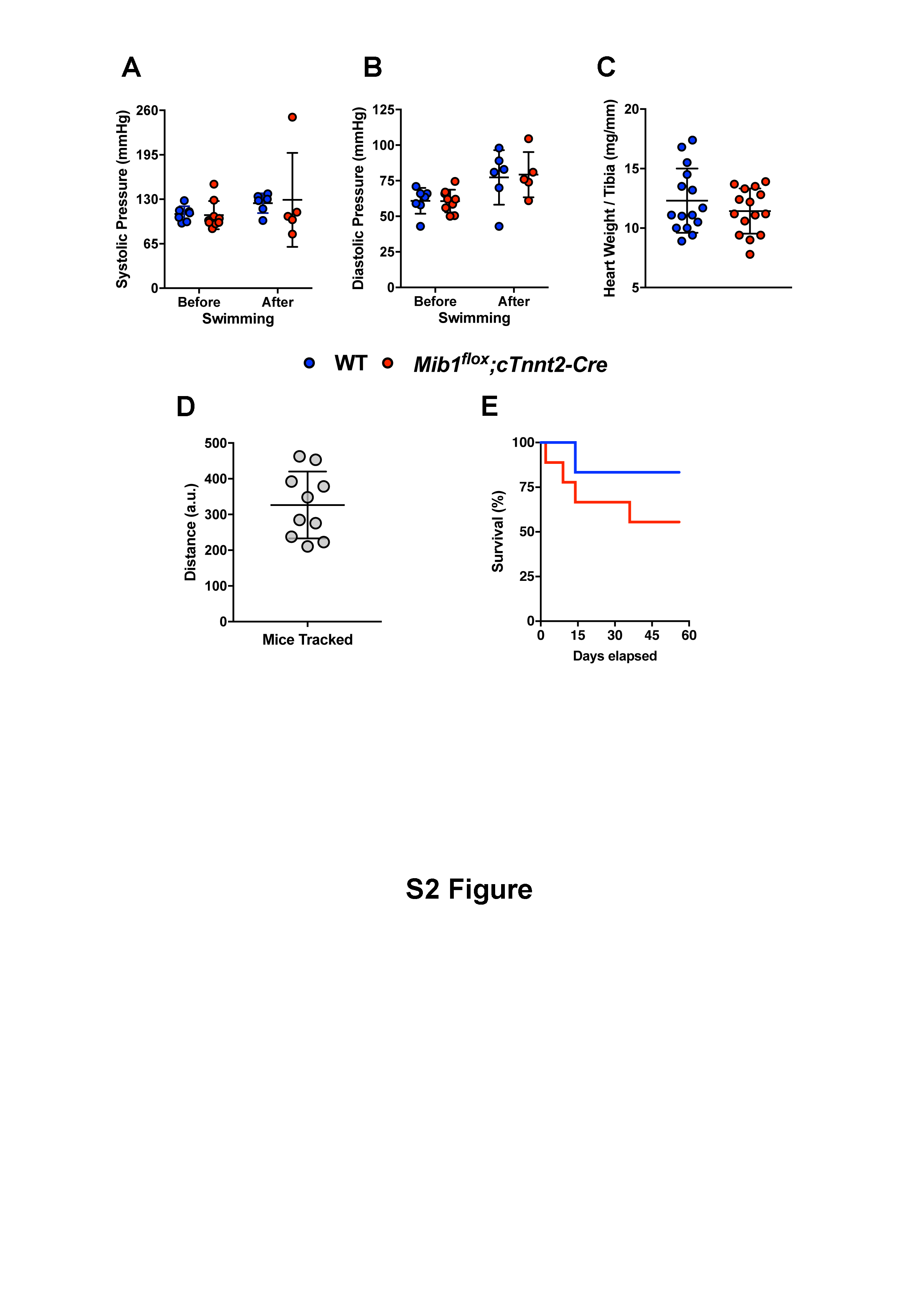

Supplement: S2 Fig — Averaged systolic pressure (A) and diastolic pressure (B) of WT and Mib1flox;Tnnt2Cre mice did not show differences between groups neither before and after swimming endurance training. Statistical significance was determined by ANOVA followed by the Tukey post-hoc test for multiple comparisons. Results are expressed as mean±SD of 7–6 WT and 9–5 Mib1flox;Tnnt2Cre mice. (C) Ratio Heart Weight/Tibia. Summary data showing no differences between WT and Mib1flox;Tnnt2Cre mice. Statistical significance was determined by unpaired two-tailed Student’s t-test. Results are expressed as mean±SD of 15 WT and 15 Mib1flox;Tnnt2Cre mice. (D) Homogeneity of training intensity. Analysis of the distance swum determined from consecutive time-lapse images for 1 min video recorded (online video 1 and 2, the dots and lines represent each animal and lines represent the tracking of a single animal.). The data is adjusted to a Gaussian pattern (passed the D’Agostino & Pearson normality test, alpha = 0.05). (E) Survival curve of mice during endurance training. Analysis of WT and Mib1flox;Tnnt2Cre mice survival percentage during the endurance swimming. (TIF) [file pone.0314840.s002.tif]

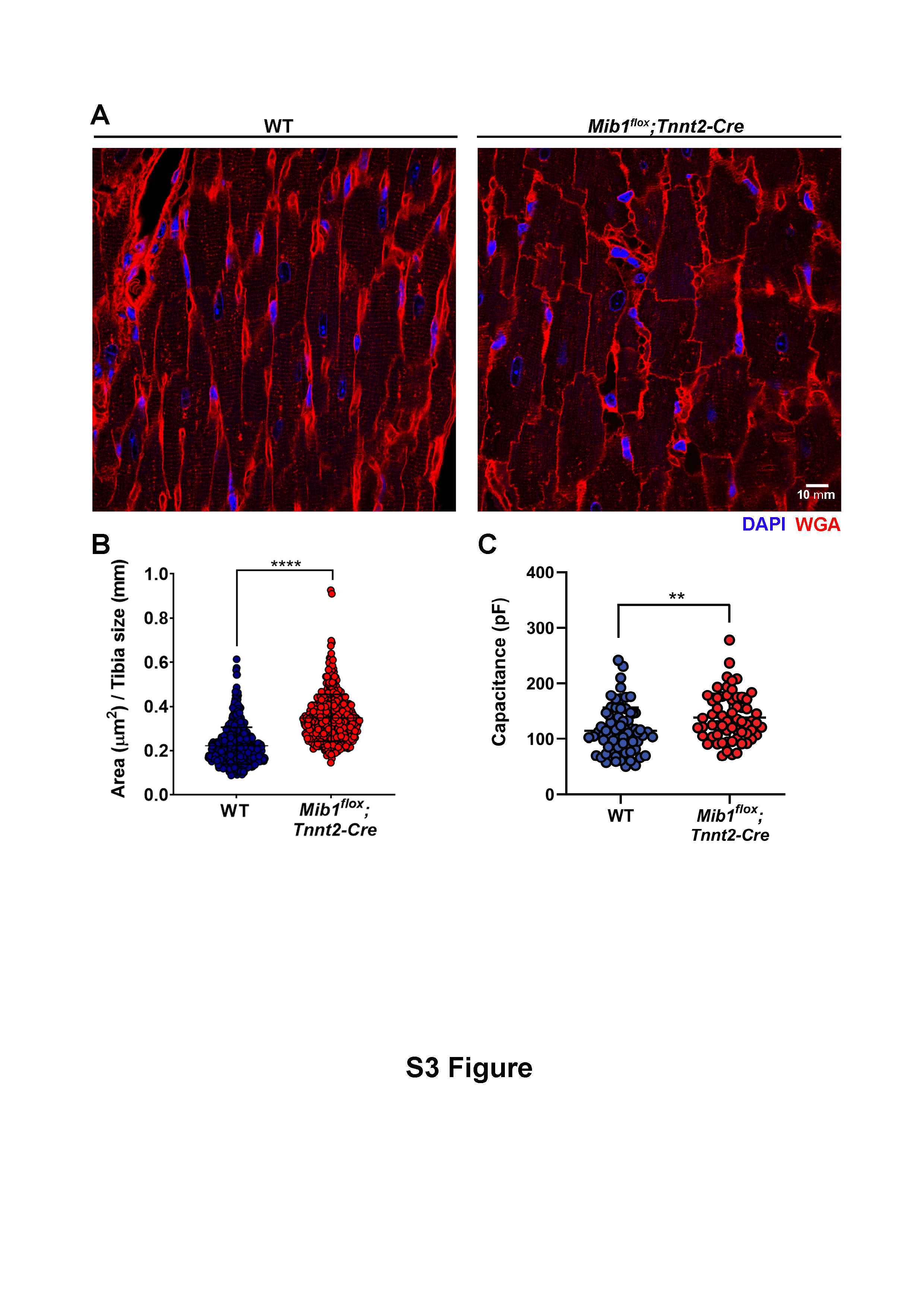

Supplement: S3 Fig — (A) Representative confocal image of myocardial sections exhibiting increased cardiomyocyte size in the Mib1flox;Tnnt2Cre mice. (B) Summary data showing that cardiomyocytes area (µm2)/ tibial (mm) ratio is increased in Mib1flox;Tnnt2Cre mice. (C) Summary data showing that cardiomyocyte capacitance is increased in Mib1flox;Tnnt2Cre mice. Statistical significance was determined by unpaired two-tailed Student’s t-test. To take into account repeated sample assessments, data were analysed with multilevel mixed-effects models. **P < 0.01; ****P < 0.0001 vs WT. In B, results are expressed as mean±SD of 400–600 cells from 3 animals per genotype. In C results are expressed as mean±SEM of 55–60 cells from 5 animals per genotype. (TIF) [file pone.0314840.s003.tif]

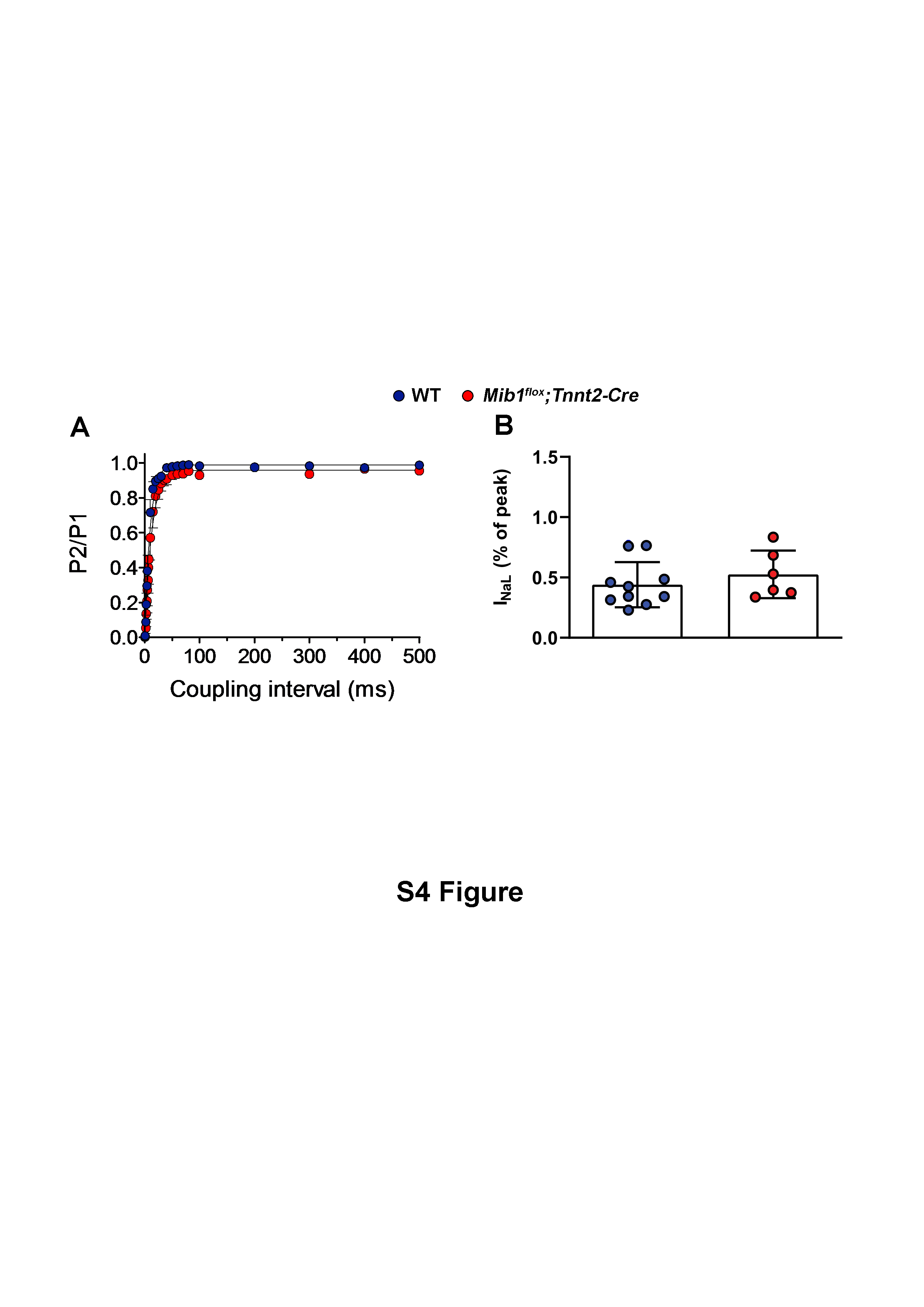

Supplement: S4 Fig — (A) Time course of peak INa recovery from inactivation measured by using a double-pulse protocol (see supplementary methods) in WT and Mib1flox;Tnnt2Cre mice. Continuous lines represent the fit of a monoexponential function to the data. Each point represents the mean±SEM of n experiments/cells. (B) INaL (expressed as percentage of the peak current) recorded at the end of 500-ms pulses to -40 mV from a holding potential of -120 mV. Unpaired two-tailed Student’s t-test was used. Statistical significance was confirmed by using non-parametric tests (two-sided Wilcoxon’s test) for small-size samples (n<15). To take into account repeated sample assessments, data were analyzed with multilevel mixed-effects models. Each bar represents the mean±SEM of 10 WT and 6 Mib1flox;Tnnt2Cre cardiomyocytes dissociated from 5 animals per genotype. (TIF) [file pone.0314840.s004.tif]

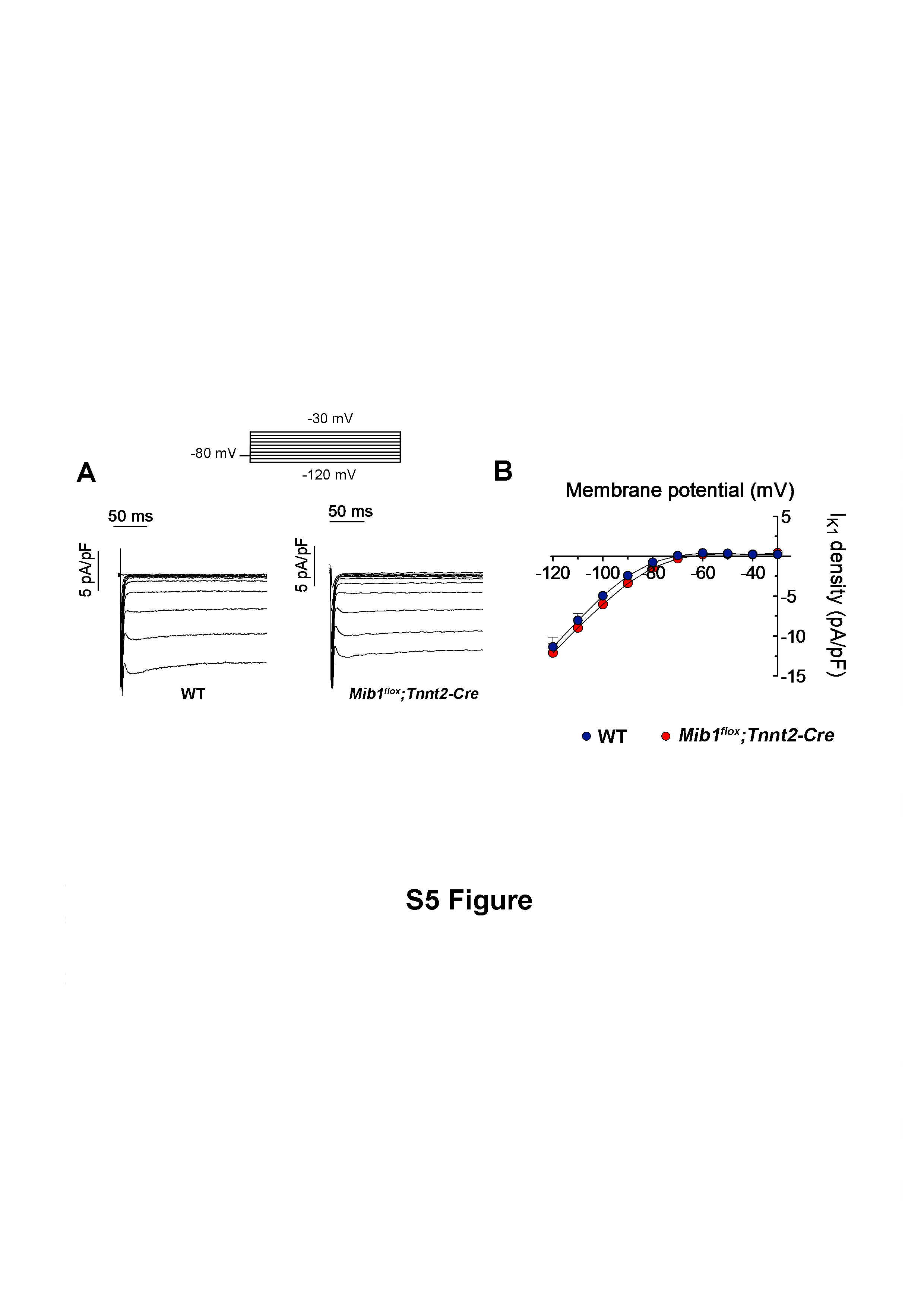

Supplement: S5 Fig — (A) Representative IK1 traces recorded in two cardiomyocytes from WT or Mib1flox;Tnnt2Cre mice by applying the pulse protocol shown at the top. (B) Mean current density-voltage curves for IK1 recorded in cardiomyocytes from both mouse groups. Unpaired two-tailed Student’s t-test was used. Statistical significance was confirmed by using non-parametric tests (two-sided Wilcoxon’s test) for small-size samples (n<15). To take into account repeated sample assessments, data were analysed with multilevel mixed-effects models. Results are expressed as mean±SEM of 19 WT and 14 Mib1flox;Tnnt2Cre cardiomyocytes dissociated from 5 animals per genotype. (TIF) [file pone.0314840.s005.tif]

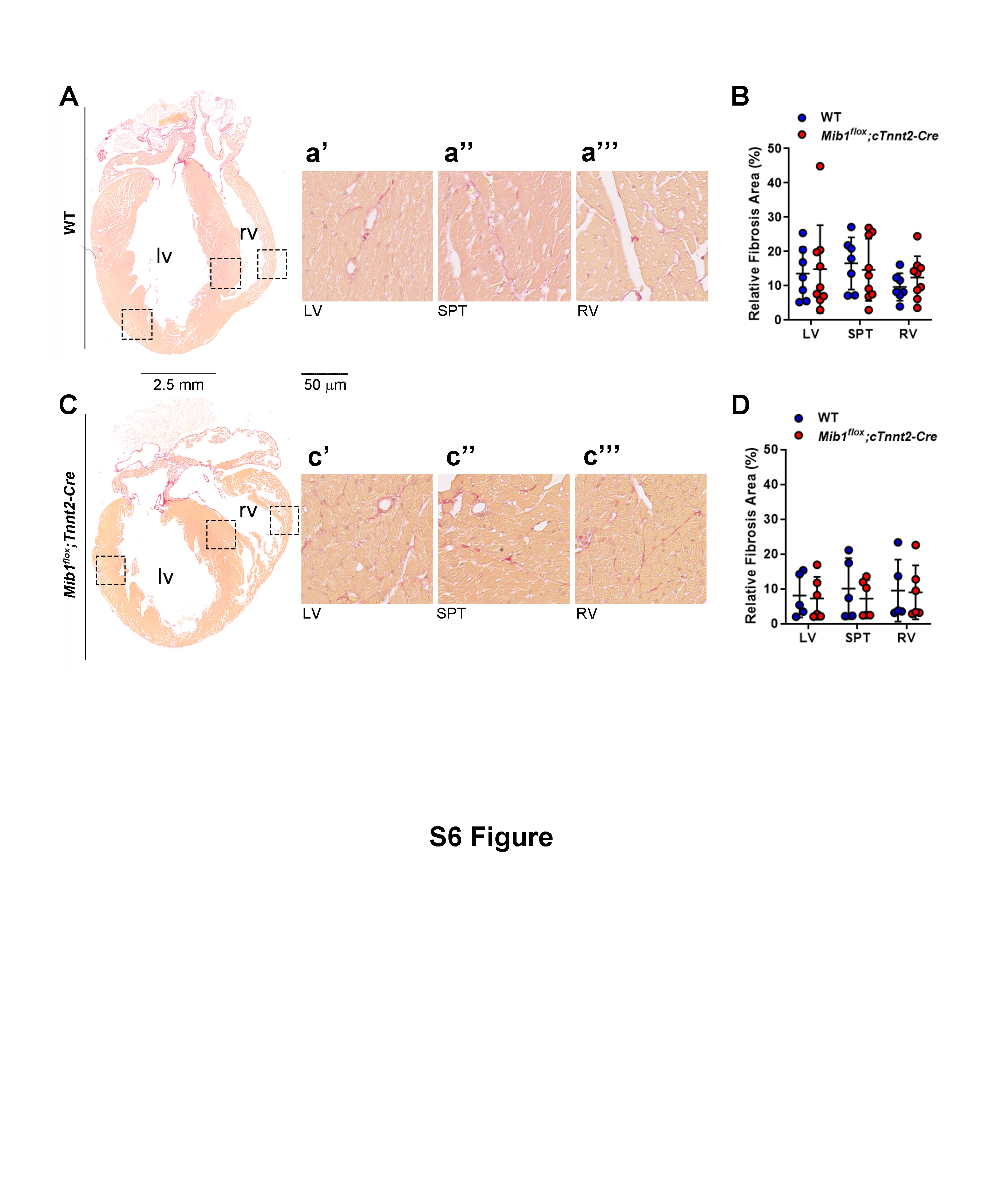

Supplement: S6 Fig — Picrosirius red observed under bright field microscopy in WT (A) and Mib1flox;Tnnt2Cre (C) heart section did not exhibit myocardial fibrosis. (a’, a,” a”’) close-up views of the LV, RV, and septum of the WT heart section. (c’, c,” c”’) close-up views of the LV, RV, and septum of the Mib1flox;Tnnt2Cre heart section. (B and D) Quantification of the areas occupied by picrosirius-positive collagen in WT and Mib1flox;Tnnt2Cre mice after endurance swimming (B) and isoproterenol (D) protocol. Statistical significance was determined by ANOVA followed by the Tukey post-hoc test for multiple comparisons. In B and D, each point represents the mean of 3 section per animal. Results are expressed as mean±SD of 5–7 WT and 6–9 Mib1flox;Tnnt2Cre mice. (TIF) [file pone.0314840.s006.tif]
