# Supplementary material for: Cardiac electrical abnormalities in a mouse model of left ventricular non-compaction cardiomyopathy
Source: PLoS One. 2025 May 7;20(5):e0314840. doi: 10.1371/journal.pone.0314840 (PMC12058163; doi:10.1371/journal.pone.0314840)

Figure 7C Nav 1.5

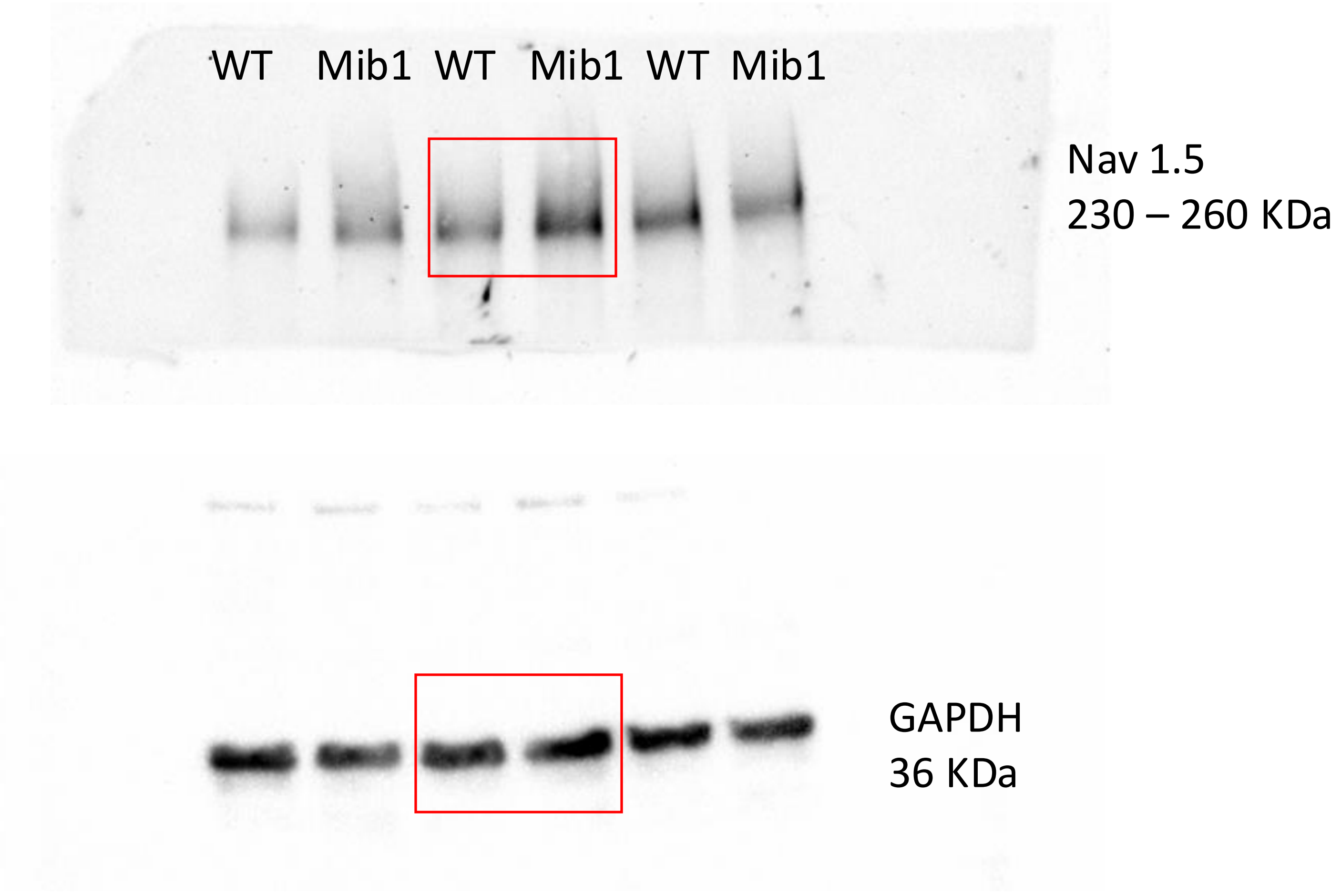

Conditions:

- 7.5% Gel
- 20ug of protein
- 1:100
- 5% milk
- 100V Electroforesis
- High MW transfer (10´)

Figure 7G

KV 4.2

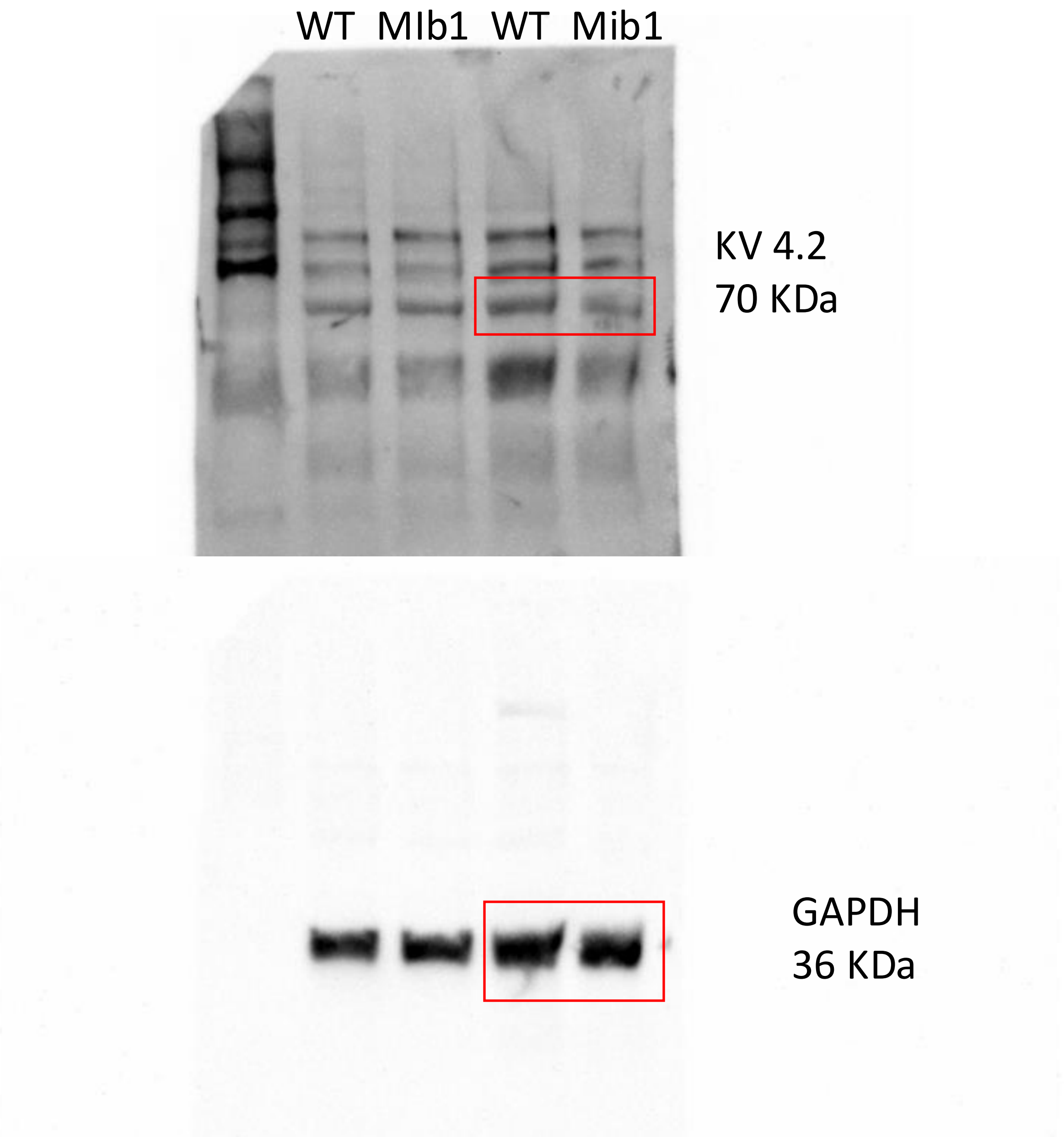

Conditions:

- 12% gel
- 40 ug protein
- 1:200
- BSA 3%

Figure 7K
KV 4.3

Conditions:
- 12% gel
- 20 ug protein
- 1:400
- 5% milk

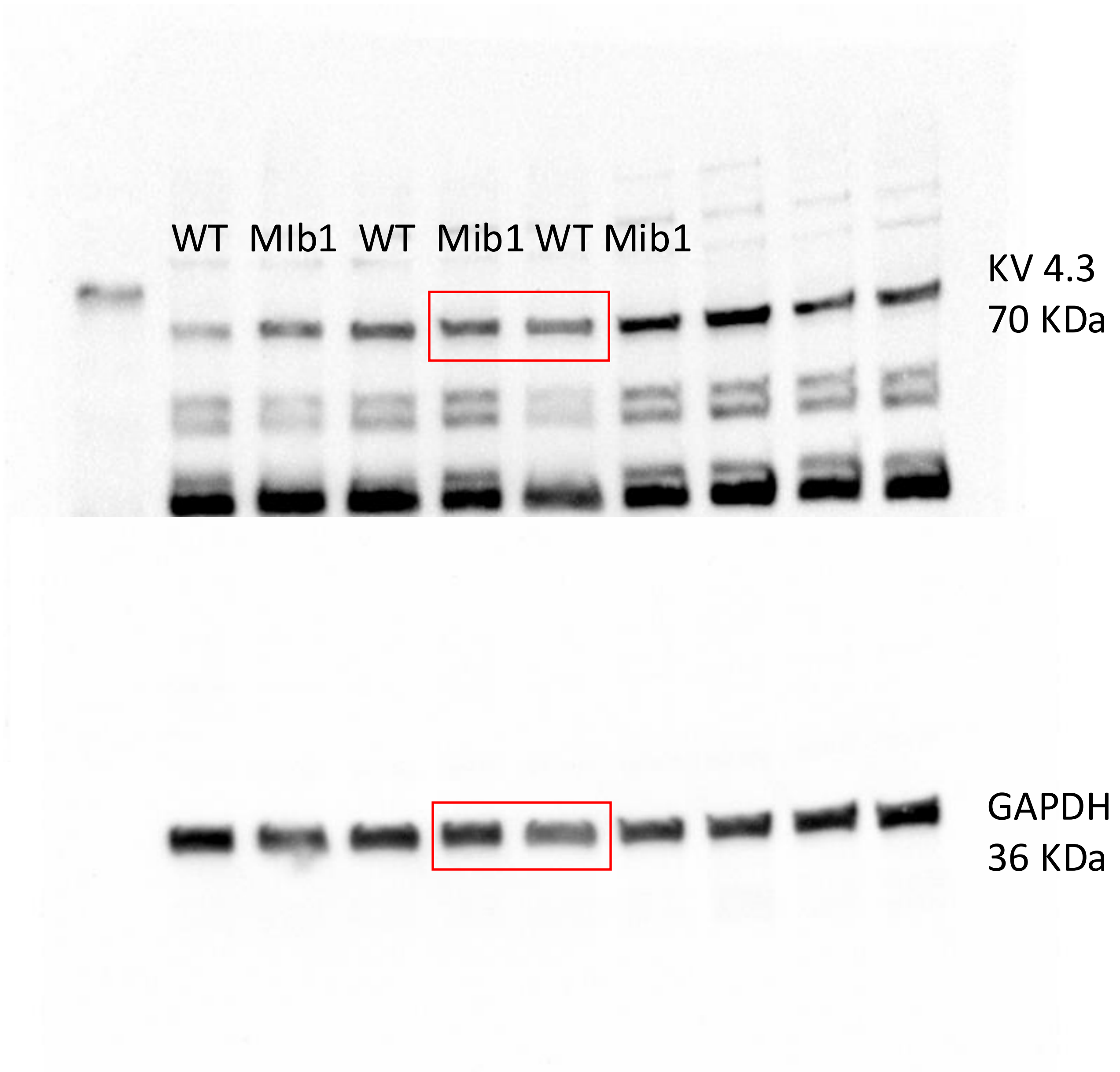

Supplement: S1 Raw images — (PDF) [file pone.0314840.s009.pdf]
